# Circulating trimethylamine N‐oxide and the risk of cardiovascular diseases: a systematic review and meta‐analysis of 11 prospective cohort studies

**DOI:** 10.1111/jcmm.13307

**Published:** 2017-08-07

**Authors:** Jiaqian Qi, Tao You, Jing Li, Tingting Pan, Li Xiang, Yue Han, Li Zhu

**Affiliations:** ^1^ Cyrus Tang Medical Institute Soochow University Suzhou Jiangsu China; ^2^ Jiangsu Institute of Haematology The First Affiliated Hospital of Soochow University Suzhou Jiangsu China; ^3^ Jiangsu Collaborative Innovation Centre of Haematology Suzhou China; ^4^ Key Laboratory of Thrombosis and Hemostasis of Ministry of Health Suzhou China; ^5^ Department of Cardiology The Second Affiliated Hospital of Soochow University Suzhou China

**Keywords:** trimethylamine N‐oxide, cardiovascular events, mortality, meta‐analysis

## Abstract

Circulating trimethylamine N‐oxide (TMAO), a canonical metabolite from gut flora, has been related to the risk of cardiovascular disorders. However, the association between circulating TMAO and the risk of cardiovascular events has not been quantitatively evaluated. We performed a systematic review and meta‐analysis of all available cohort studies regarding the association between baseline circulating TMAO and subsequent cardiovascular events. Embase and PubMed databases were searched for relevant cohort studies. The overall hazard ratios for the developing of cardiovascular events (CVEs) and mortality were extracted. Heterogeneity among the included studies was evaluated with Cochran's *Q* Test and *I* ^2^ statistics. A random‐effect model or a fixed‐effect model was applied depending on the heterogeneity. Subgroup analysis and meta‐regression were used to evaluate the source of heterogeneity. Among the 11 eligible studies, three reported both CVE and mortality outcome, one reported only CVEs and the other seven provided mortality data only. Higher circulating TMAO was associated with a 23% higher risk of CVEs (HR = 1.23, 95% CI: 1.07–1.42, *I* ^2^ = 31.4%) and a 55% higher risk of all‐cause mortality (HR = 1.55, 95% CI: 1.19–2.02, *I* ^2^ = 80.8%). Notably, the latter association may be blunted by potential publication bias, although sensitivity analysis by omitting one study at a time did not significantly change the results. Further subgroup analysis and meta‐regression did not support that the location of the study, follow‐up duration, publication year, population characteristics or the samples of TMAO affect the results significantly. Higher circulating TMAO may independently predict the risk of subsequent cardiovascular events and mortality.

## Introduction

Recent findings from studies in animal models and patients suggest that microbiota from human body may contribute to the development of various disorders, including diabetes, atherosclerosis and related cardiovascular disorders. Interestingly, trimethylamine N‐oxide (TMAO), an organic compound that was first found in marine organisms 1, 2, is recently proposed to be involved. Physiologically, TMAO functions by stabilizing proteins exposed to urea and pressure stress in deep sea creatures. In humans, choline‐, L‐carnitine‐ and lecithin‐derived mainly from red meat, egg yolks, dairy products and seafood can be hydrolysed by trimethylamine (TMA) lyase from gut flora to form the major depository of TMA in gastrointestinal tracts 3, 4. TMA is further oxygenated into TMAO by hepatic flavin monooxygenases (FMOs) in host liver, followed by kidney clearance or distribution to different tissues 5. A human mutation on FMO3 causes trimethylaminuria, a rare genetic defect with which patients fail to convert choline‐derived TMA into TMAO, resulting in a strong fishy odour from body excretions 6. So far, several strains of bacteria, including *Firmicutes*,* Proteobacteria*,* Anaerococcus hydrogenalis*,* Clostridium asparagiforme*,* C. hathewayi*,* C. sporogenes*,* Escherichia fergusonii*,* Proteus penneri*,* Providencia rettgeri*,* Edwardsiella tarda* and *Desulfovibrio desulfuricans*, have been identified as potential producers of TMAO *in vivo* 6. Recently, increased serum TMAO level is observed in patients with congestive heart failure and associated with an increased mortality in the same population 2, 7. Moreover, a positive association between plasma TMAO level and colorectal cancer has also been reported 8.

Cardiovascular diseases (CVD), including coronary artery disease, peripheral artery disease and cerebral vascular disease, are the leading cause of mortality worldwide, which contribute to over 30% of global mortality annually 9. A hallmark of CVD is atherosclerosis that is commonly associated with a broad spectrum of risk factors including hypertension, smoking, diabetes, hyperlipidaemia and ageing 10. It has been confirmed that influence of life style, such as the dietary pattern, may significantly affect the morbidity and prognosis of CVD patients. Generally, Mediterranean diet is related to decreased CVD risk, while excessive intake of carbohydrate and high‐fat diet substantially raises the incidence of CVD 11. Notably, effects of microbiota and changes of TMAO have been involved in the underlying mechanisms contributing to the association between dietary pattern and CVD risk. Indeed, an early prospective cohort study suggested that increased serum TMAO level independently predicts major adverse cardiovascular events (MACE) in over 4000 patients undergoing elective cardiac catheterization 12. Subsequent studies demonstrated that the pernicious aspects of TMAO in atherosclerosis may include the potential interactions with inflammatory pathways, cholesterol metabolism, platelet activation and subsequent thrombosis events 13. Although subsequent cohort studies in patients with coronary acute syndrome 14 and chronic kidney disease (CKD) 15, 16, 17, 18 further confirmed the prognostic role of circulating TMAO for CVD outcome, some studies failed to detect a significant association between TMAO level and CVD prognosis 19. Therefore, in this study, we performed a systematic review and meta‐analysis of all available cohort studies to quantitatively evaluate the association between baseline circulating TMAO and subsequent cardiovascular events.

## Methods

### Search strategy

We searched the PubMed and Embase databases from the inception up to 15 January 2017 for relevant studies without language restriction. Methods used are in compliance with the PRISMA statement for reporting systematic reviews and the Meta‐analysis of Observational Studies in Epidemiology (MOOSE) guidelines 20. Supplement materials displays the PRISMA checklist. The search terms used were as follows: (((trimethylamine oxide) OR tmao)) AND ((((((((((cardiovascular disease) OR coronary heart disease) OR stroke) OR heart failure) OR myocardial infarction) OR ischaemic heart disease) OR sudden cardiac arrest) OR acute coronary syndrome) OR mortality) OR cardiovascular death). To identify additional eligible studies, we also screened the references of included papers and published meta‐analysis.

### Selection criteria

The inclusion criteria of the current systematic review and meta‐analysis were as follows: (1) studies conducted in human; (2) designed as a prospective cohort study; (3) plasma or serum trimethylamine N‐oxide level was evaluated at baseline; (4) major cardiovascular events (cardiovascular mortality, MI, cardiovascular hospitalization, revascularization and stroke) or all‐cause mortality were reported as the end‐points; (5) hazard ratios (HR) with confidence intervals (CI) were reported or could be estimated as for the association between baseline TMAO and CVEs. We excluded studies that are narrative reviews, animal experiments, case reports or present insufficient data for pooling. Studies without adjustments for confounding factor, including age, gender, eGFR, N‐proBNP or traditional CVD risk factors, were also excluded. The database searching and subsequent review of the literatures were performed *via* two independent reviewers. Consultation with a senior investigator was carried out with consensus to resolve any remaining discrepancies.

### Data extraction

Surname of the first author, country of origin, year of publication, sample size, research design, gender, age, follow‐up period, serum TMAO, disease outcome, adjustment variables, HRs (or ORs, RRs) with their corresponding 95% CIs were extracted for each potentially included study. Outcome assessed in the meta‐analysis included risk of CVD, all‐cause mortality and major adverse cardiac and cerebrovascular event (MACCE) including congestive heart failure (CHF), MI, death due to cardiac causes, stroke and cardiac transplantation. Two independent investigators extracted the data using a pre‐designed data extraction form. Divergences were resolved by consensus or consulting a senior investigator.

### Quality evaluation

Newcastle–Ottawa Scale 21 was applied to evaluate the risk of bias for included studies based on study group selection, group comparability and ascertainment of exposure or outcome. We also assessed the quality of evidence in GRADE system by Grade Pro 4.04 (designed by Grade Working Group) and the results are displayed in Table S2. Two reviewers independently assessed the quality of each study.

### Statistical analysis

HRs and their corresponding Cis were used to evaluate the risk of cardiovascular outcome for patients with the highest quartile as compared with the lowest quartile of TMAO levels. The definitions of quartiles of baseline TMAO were in consistent with the original definition of the literatures. Heterogeneity among studies was evaluated using the Cochran's *Q* test and quantified using the *I* ^2^ static. Random‐effects model was applied for all meta‐analyses in spite of those with fewer than five studies, for which both fixed‐effect and random‐effects models were used to calculate pooled HRs. Fixed‐effect model was also used when heterogeneity was absent (*I* ^2^ = 0%). We explored potential sources of heterogeneity *via* subgroup analysis by study population, sample type, country of origin, follow‐up duration and year of publication. A meta‐regression model fitted with co‐variables including study population, sample type, country of origin, follow‐up duration and year of publication was analysed to explore potential sources of heterogeneity. In sensitivity analysis, the influence of each single study on overall estimates was evaluated. To assess the influence of new studies on overall effects, we performed cumulative meta‐analysis according to the year of publication for individual studies. Publication bias and selective reporting were examined by visual inspection of the asymmetry of funnel plot, in which the standard error of log HR was plotted against HR. Egger's and Begg's linear regression tests were conducted to determine statistical significance 22. The trim‐and‐fill approach was utilized to determine the number of additional studies required to overcome potential bias and provide adjusted effects. All statistical analyses were performed with STATA software, version 14.1 (StataCorp LP, College Station, TX, USA). A two‐sided *P* value <0.05 was considered with statistical significance.

## Results

### Literature search

Totally, 400 records from Embase and PubMed databases were retrieved. After removing 160 duplicates, the remaining 240 records were further examination for the titles and abstracts. Among these papers, 207 are not associated with the theme and six are not prospective studies. Full‐text review further excluded 16 articles, yielding a sum of 11 studies for the meta‐analysis.

**Figure 1:**
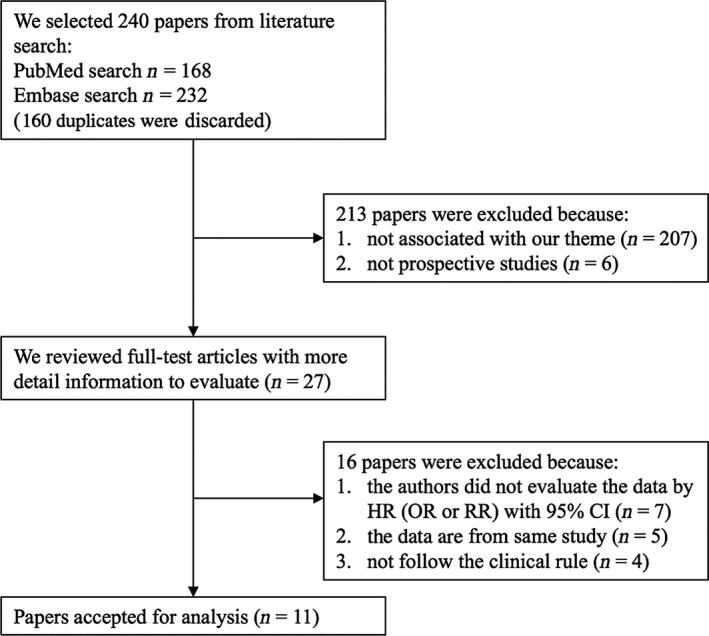
Flow chart of study selection.

Of these studies, five presented odds ratio (OR) 14, 23, 24, 25, 26 rather than hazard ratio; two did not present 95% CI 19, 27; one defined onset of chronic kidney disease as the primary outcome 28; two reported outcome by per S.D. increment of TMAO 16, 19. The studying subjects in six studies conducted by a same group were those who underwent diagnostic coronary angiography (CAG) 4, 12, 29, 30, 31, 32. Of them, five later studies, which enroled patients with heart failure (HF), chronic kidney disease (CKD), type 2 diabetes mellitus (T2DM), PAD and stable CAD, were extended subgroup follow‐ups from the first large cohort with overlaps in studying population and were thus not considered for pooling outcome in our meta‐analysis 4, 29, 30, 31, 32. Comprehensive full‐text reviewing eventually yielded 11 eligible studies for pooling in the meta‐analysis 12, 28, 33, 34, 35, 36, 37, 38, 39, 40, 41. A flow chart describing the process of study selection is presented in Figure 1.

### Study characteristics

The detailed characteristics of the 11 prospective studies published from 2013 to 2017 are shown in Table 1. Among these studies, three were conducted in the United States 12, 39, 40, while others were performed in New Zealand 41, Canada 37, Germany 36, Sweden 28, England 33, 34 and Norway 35, 38, respectively. Follow‐up durations of these studies ranged from 0.5 to 6.1 years. Patients undergoing elective coronary angiography (CAG) 12, patients with coronary artery disease (CAD) 33, 35, 41, patients with HF 34, 38, 39 and patients with CKD 28, 37, 40 were included. A study presented outcome for CAP patients with or without comorbidity of CAD 36, and this study was treated as two cohorts. Generally, the size of study cohorts varied from 112 to 4007 participants, with a total of 10,245 subjects included in the final meta‐analysis. Outcome reported from these studies was overall mortality and cardiovascular events including myocardial infarction, coronary revascularization, stroke, cardiovascular hospitalization and mortality. Two of the included studies presented TMAO levels for patients with events and without events, respectively 12, 36. All included studies were of moderate‐to‐high quality, as indicated by individual NOS scores ranging from 5 to 8.

**Table 1:** Characteristics of included prospective cohort studies

Author	Year	Country	Population	Scale	Follow‐up years	Subjects	Male (%)	Age	Assay	Sample	Design	TMAO level	Outcome	Quality score	Quality of evidence
Tang, W. H.	2013	USA	GP	Single‐centre	3	4007	64	63 ± 11	LCMS	Plasma	PCS	<2.43 *versus* >6.18 μM	CVEs or Death	8	High
Lever, M.	2014	New Zealand	CAD and CAD plus diabetes	Single‐centre	5	396	73	68 (55–93)	LCMS	Plasma	PCS	<2.8 *versus* >12.0 μM	CVEs or Death	5	Low
Kaysen, G. A.	2015	USA	CKD	Multi‐centre	5	235	55	61.8 ± 14.2	LCMS	Serum	PCS	<27.5 *versus* >66.6 μM	CVEs or Death	5	Low
Tang, W. H.	2015	USA	HF	Multi‐centre	5	112	74	57 ± 14	LCMS	Plasma	PCS	<15 *versus* >15 μM	Death	8	High
Troseid, M.	2015	Norway	HF	Single‐centre	0.5	155	83	57 ± 11	LCMS	Plasma	PCS	<9.23 *versus* >9.23 μM	Death	6	Moderate
Kim, R. B.	2015	Canada	CKD	Multi‐centre	3	2529	63	68.2 ± 12.7	LCMS	Plasma	PCS	<20.41 *versus* >20.41 μM	CVEs	5	Moderate
Ottiger, M.	2016	German	CAP and CAP plus CAD	Multi‐centre	6.1	317	59	72 (57–82)	LCMS	Plasma	PCS	<2.3 *versus* >4.1 μM	Death	5	Low
Skagen, K.	2016	Norway	CAD	Single‐centre	1	264	69	67.6 ± 8.4	LCMS	Serum	PCS	<9.77 *versus* >9.77 μM	Death	7	High
Missailidis, C.	2016	Sweden	CKD	Single‐centre	3	179	65	55 ± 14	LCMS	Plasma	PCS	<32.2 *versus* >72.2 μM	Death	6	Moderate
Suzuki, T.	2016	England	HF	Single‐centre	1	972	61	78 (69–84)	LCMS	Plasma	PCS	<5.6 *versus* >5.6 μM	Death	5	Moderate
Suzuki, T.	2017	England	CAD	Single‐centre	2	1079	72	65 (57–77)	LCMS	Plasma	PCS	<2.9 *versus* >5.1 μM	CVEs or Death	5	Moderate

HF: heart failure; CKD: chronic kidney disease; CAD: coronary artery disease; PCS: prospective cohort study.

### TMAO and CVEs

**Figure 2:**
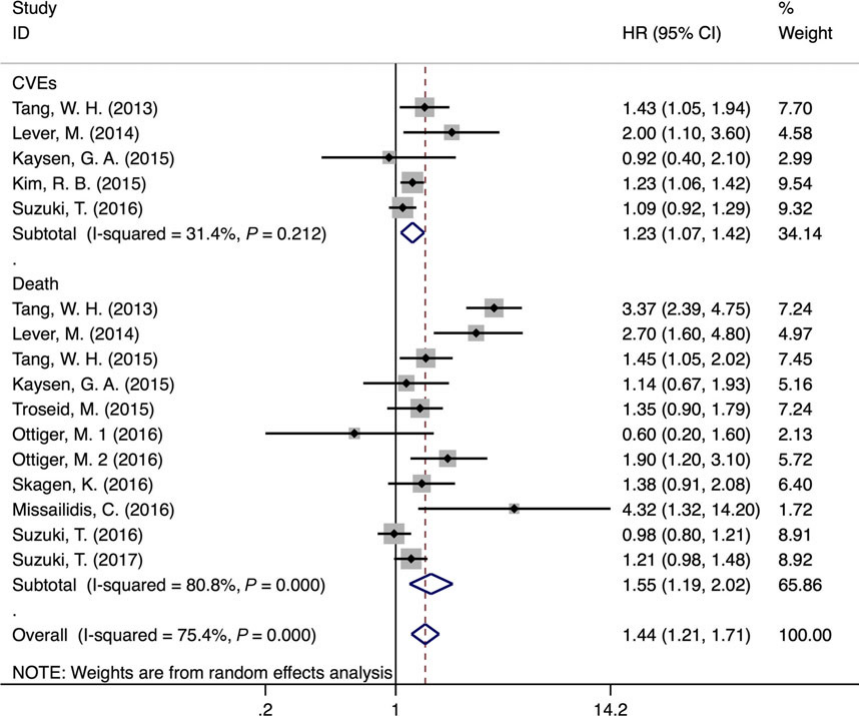
Forest plot (random‐effects model) for the association between baseline TMAO (highest *versus* lowest category) and CVD events or Death.

Five prospective cohorts with a total of 8139 participants reported HRs for the association between baseline TMAO and CVEs risk 12, 33, 37, 40, 41. Pooled results indicated that increased TMAO level was independently associated with increased risk for CVEs (HR 1.23, 95% CI: 1.07–1.42). The heterogeneity among the included studies was mild (*I* ^2^ = 31.4%, *P* = 0.212; Fig. 2). No significant publication bias was detected with the visual inspection of the funnel plot (Figure S1) or the results of Egger's (*P* = 0.497) and Begg's tests (*P* = 0.806).

### TMAO and all‐cause mortality

Ten out of 11 included studies, which included 7716 participants, reported the potential association between baseline TMAO and risk of all‐cause mortality 12, 28, 33, 34, 35, 36, 38, 39, 40, 41. Meta‐analysis demonstrated that elevated TMAO levels were independently associated with increased mortality risk (HR 1.55, 95% CI: 1.19–2.02). However, a considerable heterogeneity was noticed among different studies (*I* ^2^ = 80.8%, *P* < 0.001) (Fig. 2). Subsequently, we performed subgroup analysis to evaluate the potential effects of study population, sample type, country of origin, follow‐up years and time of publication on the association between baseline TMAO and mortality risk. We found that higher TMAO levels were associated with a 61% (HR = 1.61, 95% CI: 1.15–2.26, *I* ^2^ = 66.7%) increase of overall mortality in CAD patients, but not in patients with CKD or HF (CKD: HR = 1.99, 95% CI: 0.55–7.20, *I* ^2^ = 75.2%; HF: HR = 1.21, 95% CI: 0.93–1.57, *I* ^2^ = 60.4%; Figure S2). For the latter two subgroups, fixed‐effect model was used to estimate pooled HRs (USA: HR = 1.95, 95% CI: 1.57–2.42; CKD: HR = 1.53, 95% CI: 1.21–1.94). Furthermore, pooled HR from studies performed in the United States (HR = 1.80, 95% CI: 0.94–3.46) was not statistically significant while those in other countries showed a 41% higher risk of death in patients with increased TMAO levels (HR = 1.41, 95% CI: 1.09–1.82, *I* ^2^ = 68.8%). In addition, elevated TMAO was associated with increased mortality risk in studies with plasma samples (HR = 1.63, 95% CI: 1.19–1.23, *I* ^2^ = 84.5%), but not in studies with serum samples (HR = 1.28, 95% CI: 0.93–1.78, *I* ^2^ = 0%). Additional subgroup analyses showed similar increases of mortality risk in studies with different follow‐up periods (follow‐up <5 years: HR = 1.59, 95% CI: 1.09–2.33, *I* ^2^ = 87.8%; follow‐up >5 years: HR = 1.53, 95% CI: 1.07–2.19, *I* ^2^ = 56.9%). As all included studies in this meta‐analysis have been published within 5 years, we checked whether the year of publication affected the pooled HR by cumulative analysis (Figure S3a, 3b). The association of baseline TMAO and mortality risk remained after the addition of more recent studies, which was further confirmed by the results of meta‐regression for publication year (*P* = 0.285). Fitting other variables, including country of origin, follow‐up years, sample types and population, into the meta‐regression model did not indicate additional sources of heterogeneity (Table 2). Results of sensitivity analysis did not support a certain study impacting the overall outcome significantly (Figure S4a, 4b). According to the results, no individual study was found responsible for the observed heterogeneity.

**Table 2:** Stratified analyses of pooled hazard risks of TMAO and CVD events

Stratified analysis	Number of studies	Pooled HR (95% CI)	Heterogeneity	Meta‐regression (*P* value)
Country				0.503
USA	3	1.80 (0.94–3.46)	*Q* = 16.85, *P* = 0.000, *I* ^2^ = 88.1%	
Non‐USA	8	1.41 (1.09–1.82)	*Q* = 22.45, *P* = 0.002, *I* ^2^ = 68.8%	
Follow‐up years				0.858
<5 years	6	1.59 (1.09–2.33)	*Q* = 41.14, *P* = 0.000, *I* ^2^ = 87.8%	
≥5 years	5	1.53 (1.07–2.19)	*Q* = 9.28, *P* = 0.054, *I* ^2^ = 56.9%	
Public year				0.285
Before 2015	6	1.83 (1.20–2.78)	*Q* = 22.10, *P* = 0.000, *I* ^2^ = 81.9%	
After 2015	5	1.36 (0.89–2.06)	*Q* = 13.62, *P* = 0.009, *I* ^2^ = 70.6%	
Population				0.213
HF	3	1.28 (1.11–1.47)	*Q* = 0.80, *P* = 0.670, *I* ^2^ = 0%	
CAD	5	1.47 (1.06–2.02)	*Q* = 10.71, *P* = 0.030 *I* ^2^ = 62.6%	
CKD	4	1.58 (1.13–2.19)	*Q* = 4.82, *P* = 0.186 *I* ^2^ = 37.7%	
Samples				0.535
Plasma	9	1.63 (1.19–2.23)	*Q* = 51.63, *P* = 0.000, *I* ^2^ = 84.5%	
Serum	2	1.28 (0.93–1.78)	*Q* = 0.31, *P* = 0.577, *I* ^2^ = 0%	

**Figure 3:**
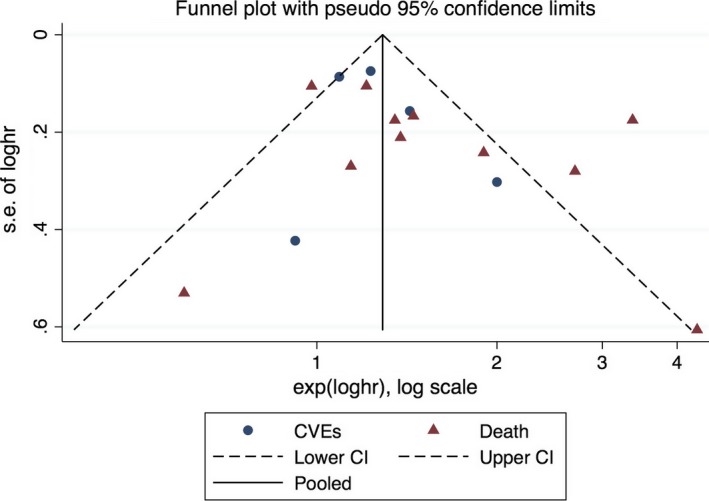
Funnel plot for the association between baseline TMAO (highest *versus* lowest category) and CVD events or Death.

**Figure 4:**
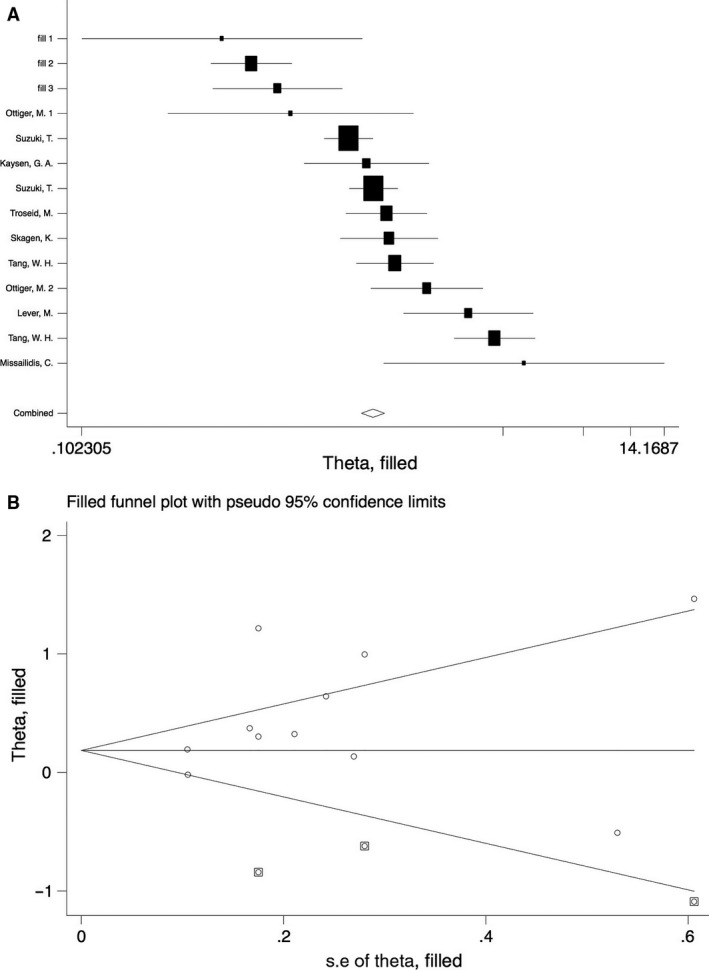
Meta trim‐and‐fill model (**A**) and filled funnel plot (**B**).

The funnel plot showed obvious asymmetry, indicating the presence of publication bias (Fig. 3). Application of trim‐and‐fill model suggested that three more studies may be required to eliminate the publication bias, although the pooled effect was markedly different (HR = 1.23, 95% CI: 0.90–1.67) (Fig. 4A and B). Nonetheless, statistics using the Egger's (*P* = 0.436) and Begg's (*P* = 0217) regression tests did not show significant bias in publication (Figure S5). We also use the standard ‘Risk of bias’ tool and Newcastle‐Ottawa Scale (NOS) to evaluate bias (Figure S6a, S6b; Tables 1 and S3).

## Discussion

In this study, by pooling the data from 11 available prospective cohort studies incorporating 10,245 participants, we found that higher circulating TMAO at baseline was independently associated with 23% increment risk of subsequent CVEs and 55% increment of all‐cause mortality. Although significant heterogeneity was detected for the association of baseline TMAO and risk of all‐cause mortality, subsequent subgroup and meta‐analyses did not support that the characteristics of included patients, follow‐up duration, location of the study and sample of TMAO had significant effects on the association between TMAO and CVEs or mortality risks. These results suggest that participants with higher baseline TMAO were at higher risk of future cardiovascular risk. Future studies are needed to determine whether circulating TMAO confers prognostic efficacy for CVDs.

Intake of foods such as eggs, beef and fish is known to raise the blood and urine concentrations of TMAO 7, 42. Increased TMAO has been demonstrated to promote insulin resistance and adipose inflammation in mice and associated with increased risk of developing type 2 diabetes mellitus in human 29. More recently, TMAO has been suggested as a candidate of proatherogenic molecules in circulation due to its detrimental metabolic effects and contribution in foam cell formation 21. In addition, the interference of bile acid metabolism and sterol transportation has also been shown to underlie the pernicious roles of TMAO in the development of atherosclerosis 7. As mentioned previously, a pilot study suggests the potential predictive role of circulating TMAO as risk of CVEs in patients undergoing elective coronary angiography 12. Subsequently, higher high‐TMAO level was linked to adverse clinical outcome in patients with HF, CKD and T2DM, respectively 29, 32, 39. Subsequent analyses were not always supportive of the predictive role of TMAO.

Nevertheless, the results on the link between TMAO and CVD remain limited and inconsistent. For instance, TMAO exhibited no significant predictive value on CVEs and mortality in a 4‐year observational cohort including 235 patients with CKD 40. Similar result was obtained from a more recent U.K. study in patients with HF 19. Two studies from Norway enrolling patients with CAD and HF also failed to show any evident correlation between TMAO and risk of death 38, 43. In another German study recruiting patients with comorbidities of community‐acquired pneumonia and CAD, higher TMAO level did not sufficiently predict an increase in mortality rate 36. More intriguingly, consumption of seafood that is rich in TMAO has been found to confer significant protection against cardiovascular disease rather than predisposing to deleterious outcome. To the best of our knowledge, ours is the first systematic review and meta‐analysis exploring the prognostic value of TMAO in CVEs and mortality has been published so far. Results of our study further confirmed that higher TMAO at baseline was associated with increased risk of CVEs and all‐cause mortality. Moreover, our subsequent subgroup analyses found that the vast disagreement among studies may attribute to various aspects, including patient population (CKD, HF, T2DM and CAD), race (Black and White) and sample type (serum and plasma). Spectacularly, only studies conducted outside the United States exhibited a significant overall HR for the high‐TMAO effect on mortality. In spite, studies published after the year 2015 did not suggest a positive correlation between increased TMAO and higher risk of death. This finding is consistent with our cumulative analysis that showed a progressive reduction in the pooled HR with the addition of more recent studies. This discrepancy may be resulted from different followed‐up durations. However, this was not supported by our meta‐regression for publication time and follow‐up duration. Of note, pooled HRs of mortality was substantially different between studies using plasma and those testing serum samples. This finding was supported by the significant difference between baseline TMAO levels in plasma and serum indicated in a methodology study. However, the result remains uncertain given limited number of studies in the serum group, and further studies with larger sample size are needed to confirm our results.

The mechanisms underlying the potential association between TMAO and risks of cardiovascular diseases deserve further investigation. Some previously published experimental studies may provide some clues. TMAO may increase platelet activity through the potentiation of cytoplasmic calcium release, by which it may predispose to a hyper‐coagulating status and increased thrombotic events 13. Additional mechanisms by which TMAO regulates cardiovascular health may involve the prolongation of angiotensin effects, which is also likely to exacerbate cardiac remodelling and contributes to detrimental outcome in heart failure 44. TMAO is also known to play a role in the perturbation of metabolic networks in type 2 diabetes, in which a synergistic effect by increased TMAO on the prognosis has been reported 29. In patients with compromised renal function, attenuated clearance of TMAO may results in an elevation of circulating TMAO. Subsequently increased TMAO can induce kidney fibrosis *via* activation of the TGF‐β‐Smad3 pathway 4. In addition, increased risk of having cancer may also be additive to poor prognosis in those with elevated TMAO levels 45. As kidney is the major organ responsible for the clearance of circulating TMAO in human, a drastic elevation of serum TMAO has been readily seen in those with renal failure. Consistently, plasma TMAO level was found to be negatively related the eGFR. Uraemic patient had the highest TMAO level, which can be dramatically reduced by haemodialysis. This may possible explain the very high heterogeneity observed in patients with CKD 28. Furthermore, studies that adjusted for more comprehensive CVD risk factors tend to show positive prognostic effects on mortality by TMAO 12, indicating these factors as underlying sources of inter‐study heterogeneity.

### Strength and limitations

Our study demonstrated potential prognostic power of TMAO in a variety of populations. To our knowledge, this is the first meta‐analysis that shows a positive correlation between high‐TMAO level and adverse cardiovascular outcome. In this study, we included only prospective cohorts and followed the PRISMA and MOOSE guidelines. Potential sources of heterogeneity were explored using different methods, and trim‐and‐fill strategy was applied to solve the possibility of publication bias. All of the above aspects added to the power of the study. Our study also has potential limitations, which should be considered when interpreting the results. Firstly, studies that did not provide sufficient data for pooling were excluded from the meta‐analysis 14, 23, 24, 25, 26, which may raise risk of bias in the overall effects by TMAO. Considerable risk of publication bias was identified for the association between high TMAO and all‐cause mortality, leading to a dubitative conclusion of the link. Moreover, considerable heterogeneities existed in baseline clinical characteristics including age, gender and race across individual studies. Additionally, uncontrolled cofounding factors including dietary patterns and genetic variation may significantly affect the concentration of TMAO. Inclusion of those studies without comprehensive adjustment may yield inaccurate effects. Moreover, the lack of continuous data made it difficult to draw a quantitative result on the difference of TMAO between subjects with and without events. Finally, results of our meta‐analysis of observational studies could only provide the potential temporal association between increased circulating TMAO and subsequent CVD risk. Whether increased TMAO was causative to poor cardiovascular outcome deserves further investigation.

## Conclusion

Participants with higher baseline TMAO were at higher risk for future cardiovascular risk. Further large‐scale prospective cohorts or even interventional studies are warranted to evaluate the diagnostic power of TMAO and its causative role on cardiovascular outcome.

## Conflict of interest

The authors declare no conflict of interest.

## Supporting information


**Table S1** Overview of multivariable relationships of TMAO with CVEs or Death.
**Table S2** Quality of Evidence evaluated by GRADE system.
**Table S3** Assessment of Newcastle‐Ottawa Scale.
**Fig. S1** Egger linear regression test and Begg's test plot with 95% CIs for the relationship between TMAO level and CVEs.
**Fig. S2** Forest plot (random‐effects model) for the association between TMAO (lowest vs. highest category) and CVD risk in different populations.
**Fig. S3** Cumulative analysis for baseline TMAO level and death (a) and CVEs (b).
**Fig. S4** Sensitivity analysis for TMAO level and death (a) and CVEs (b).
**Fig. S5** Egger linear regression test and Begg's test plot with 95% CIs for the relationship between baseline TMAO level and death risk.
**Fig. S6a** Risk of bias graph: review authors’ judgements about each risk of bias item presented as percentages across all included studies.
**Fig. S6b** Risk of bias summary: review authors’ judgements about each risk of bias item for each included study.
