# Supplementary material for: Circulating trimethylamine N‐oxide and the risk of cardiovascular diseases: a systematic review and meta‐analysis of 11 prospective cohort studies
Source: J Cell Mol Med. 2017 Aug 7;22(1):185–94. doi: 10.1111/jcmm.13307 (PMC5742728; doi:10.1111/jcmm.13307)
Supplement: Supplementary file 1 — Table S1 Overview of multivariable relationships of TMAO with CVEs or Death. Table S2 Quality of Evidence evaluated by GRADE system. Table S3 Assessment of Newcastle‐Ottawa Scale. Fig. S1 Egger linear regression test and Begg's test plot with 95% CIs for the relationship between TMAO level and CVEs. Fig. S2 Forest plot (random‐effects model) for the association between TMAO (lowest vs. highest category) and CVD risk in different populations. Fig. S3 Cumulative analysis for baseline TMAO level and death (a) and CVEs (b). Fig. S4 Sensitivity analysis for TMAO level and death (a) and CVEs (b). Fig. S5 Egger linear regression test and Begg's test plot with 95% CIs for the relationship between baseline TMAO level and death risk. Fig. S6a Risk of bias graph: review authors’ judgements about each risk of bias item presented as percentages across all included studies. Fig. S6b Risk of bias summary: review authors’ judgements about each risk of bias item for each included study. [file JCMM-22-185-s001.pdf]

Supplement Materials

Table S1. Overview of multivariable relationships of TMAO with CVEs or Death.

Outcome	Author	Year	Adjusted HR (95% CI)	Adjusted Covariate
CVEs	Tang, W. H.	2013	1.43 (1.05-1.94)	age, sex, smoking status, systolic blood pressure, low-density lipoprotein cholesterol level, high-density lipoprotein cholesterol level, status with respect to diabetes mellitus and log-transformed high-sensitivity C-reactive protein level.
	Lever, M.	2014	2.00 (1.10-3.60)	eGFR
	Kaysen, G. A.	2015	0.92 (0.40-2.10)	race, diabetes, and prealbumin
	Kim, R. B.	2015	1.23 (1.06-1.42)	age, sex, race and presence or absence of diabetes and cardiovascular commidities
	Suzuki, T.	2016	1.09 (0.92-1.29)	age, blood urea and eGFR
Death	Tang, W. H.	2013	3.37 (2.39-4.75)	age, sex, smoking status, systolic blood pressure, low-density lipoprotein cholesterol level, high-density lipoprotein cholesterol level, status with respect to diabetes mellitus and log-transformed high-sensitivity C-reactive protein level.
	Lever, M.	2014	2.70 (1.60-4.80)	eGFR
	Kaysen, G. A.	2015	1.14 (0.67-1.93)	none
	Tang, W. H.	2015	1.45 (1.05-2.02)	traditional CVD risk factors, log-transformed hsCRP, log-transformed eGFR, hsCRP and cystatin C
	Troseid, M.	2015	1.35 (0.90-1.79)	eGFR, CRP and NT-proBNP
	Ottiger, M.	2016	0.60 (0.20-1.60) and 1.90 (1.20-3.10)	comorbidities (coronary artery disease, congestive heart failure, cerebrovascular disease, peripheral artery occlusive disease, diabetes mellitus, chronic kidney disease, neoplastic disease, and chronic obstructive pulmonary disease)
	Skagen, K.	2016	1.38 (0.91-2.08)	age and eGFR
	Missailidis, C.	2016	4.32 (1.32-14.2)	age, gender, SGA, albumin, DM and mGFR
	Suzuki, T.	2016	0.98 (0.80-1.21)	age, blood urea and eGFR
	Suzuki, T.	2017	1.21 (0.98-1.48)	age, blood urea and eGFR

eGFR= estimated glomerular filtration rate; CRP= C-reactive protein; NT-pro-BNP= amino-terminal pro-brain natriuretic peptide; ICED score= Index of Coexisting Disease Score; ESRD= end-stage renal disease

Table S2. Quality of Evidence evaluated by GRADE system

Author	Year	Design	Downgrade quality of evidence					Upgrade quality of evidence			Quality of Evidence
			Risk of Bias	Inconsistency	Indirectness	Imprecision	Publication Bias	Large effect	PCWCE	Dose-response Gradi	
Tang, W. H.	2013	PCS	no	no	no	serious (-1)	undetected	large (+1)	no	no	High ⊕⊕⊕⊕
Lever, M.	2014	PCS	no	serious (-1)	no	serious (-1)	undetected	no	no	no	Low ⊕⊕□□
Kaysen, G. A.	2015	PCS	no	serious (-1)	serious (-1)	no	undetected	no	no	no	Low ⊕⊕□□
Tang, W. H.	2015	PCS	no	no	no	serious (-1)	undetected	large (+1)	no	no	High ⊕⊕⊕⊕
Troseid, M.	2015	PCS	no	no	no	serious (-1)	undetected	no	no	no	Moderate ⊕⊕⊕□
Kim, R. B.	2015	PCS	no	serious (-1)	no	no	undetected	no	no	no	Moderate ⊕⊕⊕□
Ottiger, M.	2016	PCS	no	serious (-1)	no	serious (-1)	undetected	no	no	no	Low ⊕⊕□□
Skagen, K.	2016	PCS	no	no	no	no	undetected	no	no	no	High ⊕⊕⊕⊕
Missailidis, C.	2016	PCS	no	no	no	serious (-1)	undetected	no	no	no	Moderate ⊕⊕⊕□
Suzuki, T.	2016	PCS	no	serious (-1)	no	no	undetected	no	no	no	Moderate ⊕⊕⊕□
Suzuki, T.	2017	PCS	no	serious (-1)	no	no	undetected	no	no	no	Moderate ⊕⊕⊕□

Table S3. Assessment of Newcastle-Ottawa Scale

Author	Year	Selction	Comparability	Outcome	Total
Tang, W. H.	2013	4	2	2	8
Lever, M.	2014	3	1	1	5
Kaysen, G. A.	2015	2	1	2	5
Tang, W. H.	2015	4	2	2	8
Troseid, M.	2015	3	1	2	6
Kim, R. B.	2015	2	1	2	5
Ottiger, M.	2016	3	1	1	5
Skagen, K.	2016	4	1	2	7
Missailidis, C.	2016	3	2	1	6
Suzuki, T.	2016	3	1	1	5
Suzuki, T.	2017	3	1	1	5

a

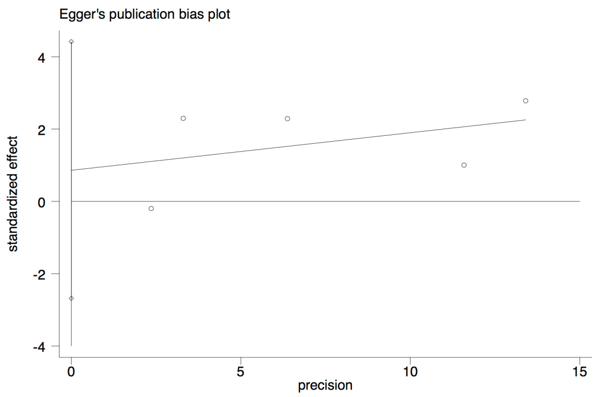

b

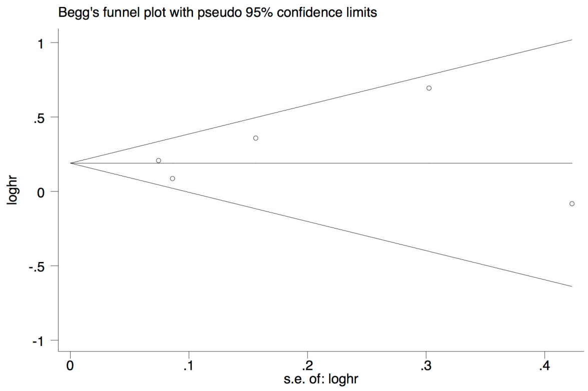

Fig. S1. Egger linear regression test and Begg's test plot with 95% Cis for the relationship between TMAO level and CVEs

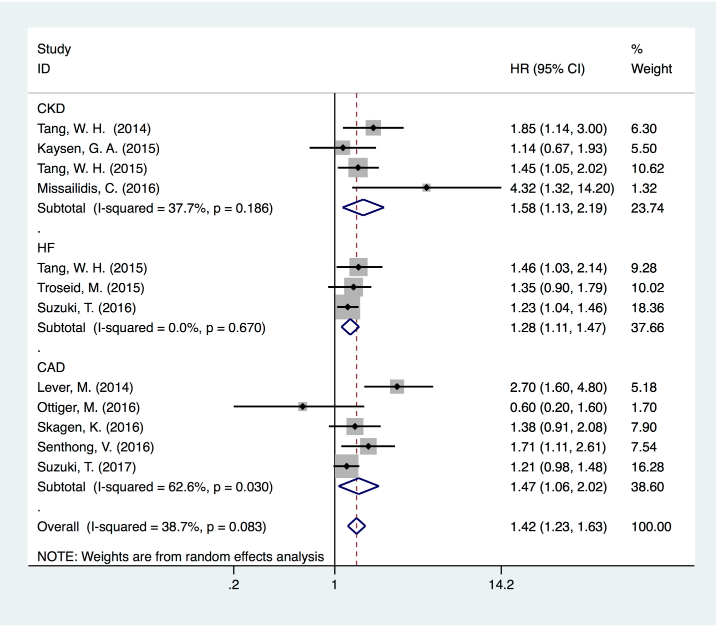

Fig. S2. Forest plot (random-effects model) for the association between TMAO (lowest vs. highest category) and CVD risk in different populations.

a

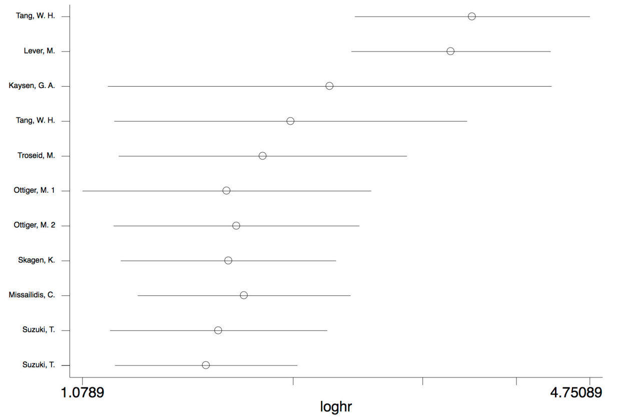

b

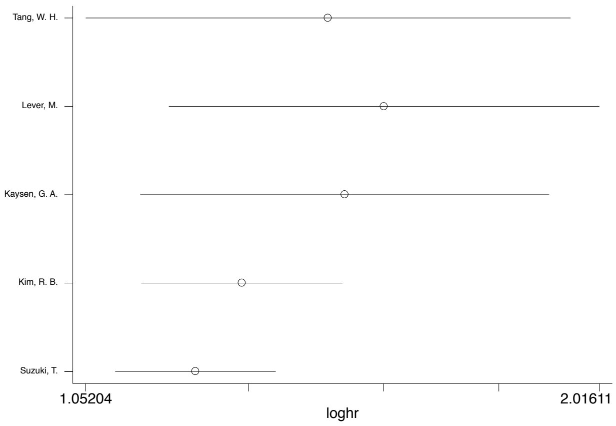

Fig. S3. Cumulative analysis for baseline TMAO level and death **(a)** and CVEs **(b)**.

a

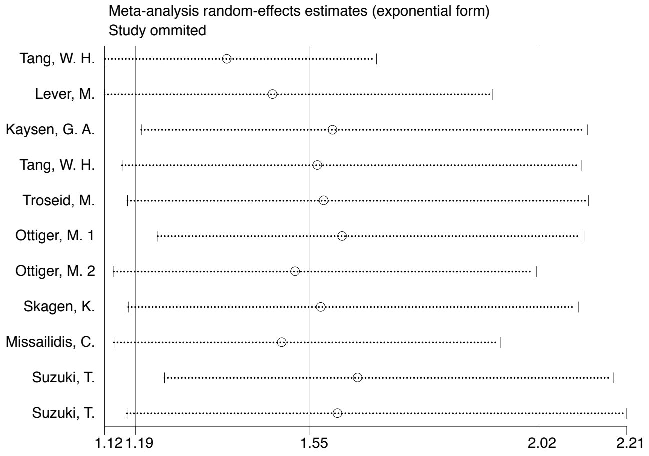

b

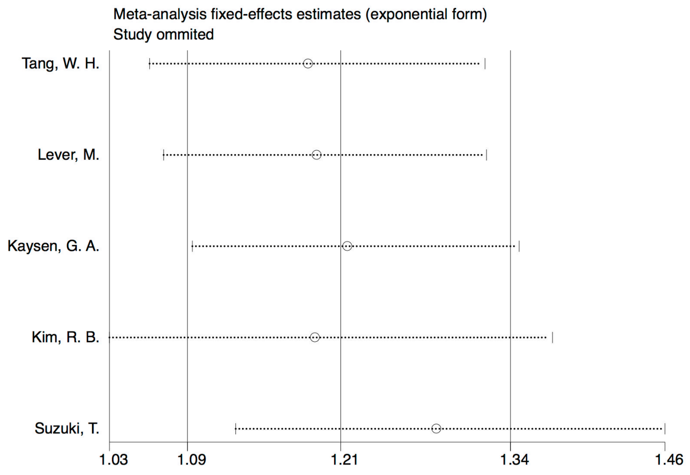

Fig. S4. Sensitivity analysis for TMAO level and death **(a)** and CVEs **(b)**.

a

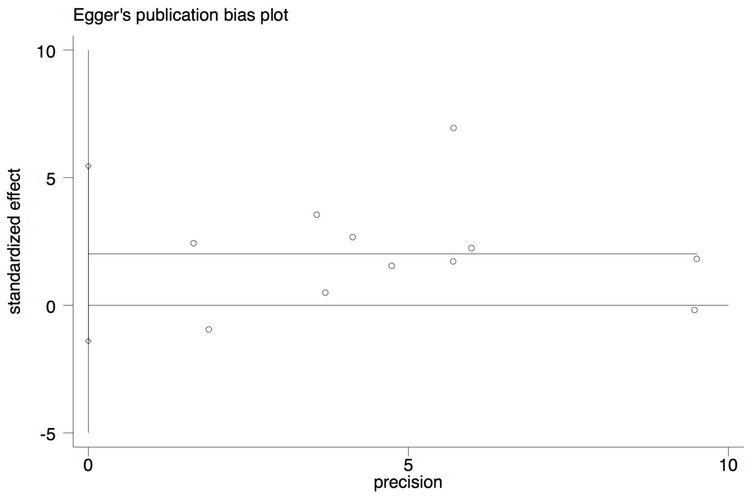

b

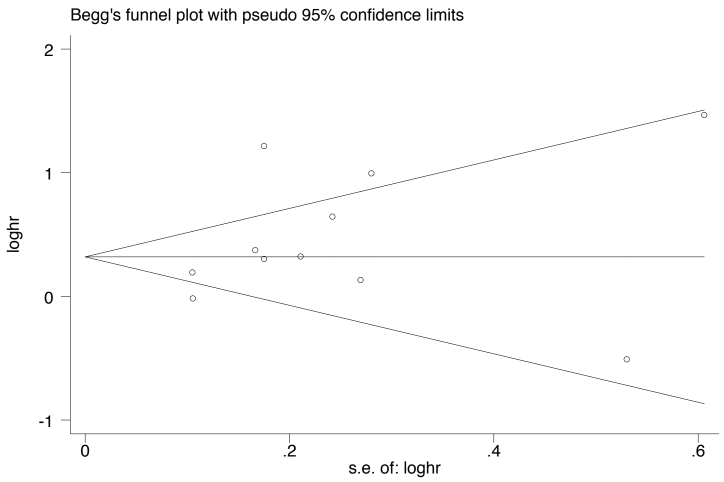

Fig. S5. Egger linear regression test and Begg's test plot with 95% Cis for the relationship between baseline TMAO level and death risk.

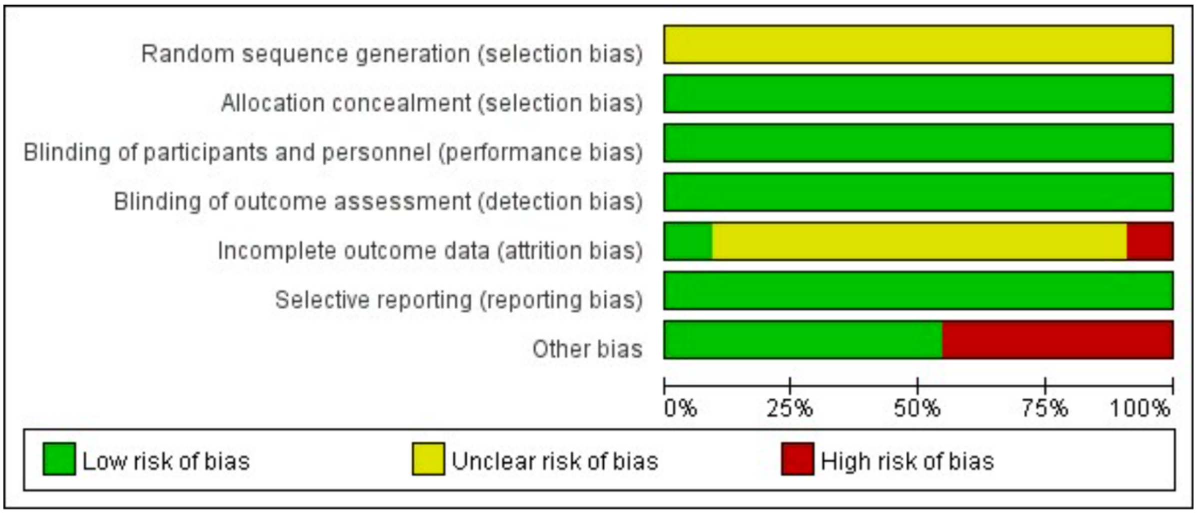

Fig. S6a. Risk of bias graph: review authors' judgements about each risk of bias item presented as percentages across all included studies.

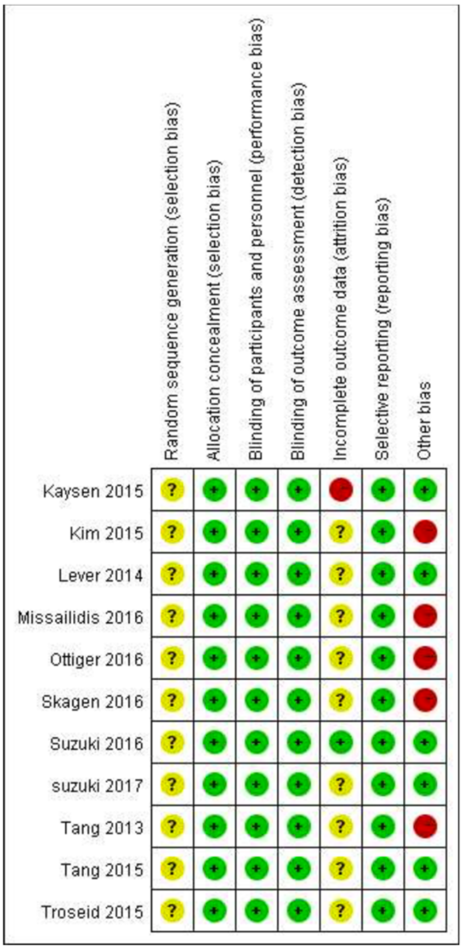

Fig. S6b. Risk of bias summary: review authors' judgements about each risk of bias item for each included study.
